# Bullous cutaneous reactions

**DOI:** 10.11604/pamj.2018.31.188.16608

**Published:** 2018-11-16

**Authors:** Mohamed Jira, Taoufik Amezyane

**Affiliations:** 1 Internal Medicine Department, Mohammed V Military Teaching Hospital, Rabat, Morocco

**Keywords:** Allopurinol hypersensitivity, bullous cataneous reactions

## Image in medicine

Allopurinol (xanthine oxidase inhibitor) is indicated for the treatment of symptomatic hyperuricemia, the treatment of gout and the treatment and prevention of uric and calcium lithiasis. It is one of the leading drugs for severe toxic dermal reactions, such as Lyell Syndrome (NET), Stevens-Johnson Syndrome (SJS) and Dress Syndrome (Drug reaction with eosinophilia and systemic symptoms). We report the case of a 45-year-old woman who was hospitalized for bullous skin rashes, without pathological history, the patient had generalized erythematous and bullous rash (A, B and C) one week after taking allopurinol, prescribed by his rheumatologist for a gout. The diagnosis of bullous Cutaneous reaction due to allopurinol was retained, allopurinol was discontinued and the course was favorable with the disappearance of cutaneous lesions.

**Figure 1:**
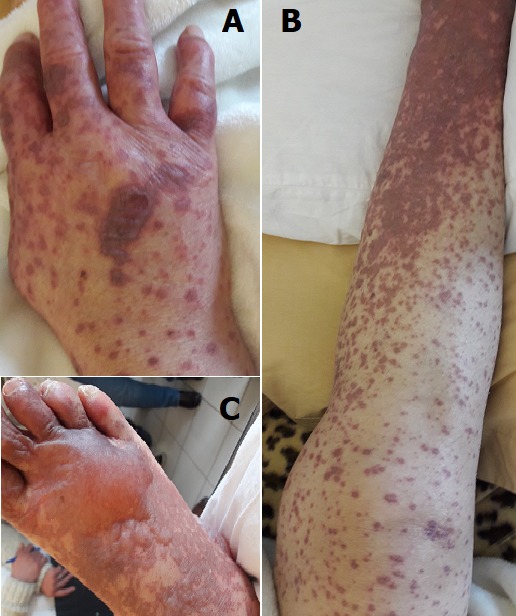
A) bullous cutaneous reaction of the hand; B) bullous cutaneous reaction of the leg; C) bullous cutaneous reaction of the foot

